# The Current State of Cell Therapies for Cerebrovascular Diseases

**DOI:** 10.1155/2016/5215824

**Published:** 2016-09-08

**Authors:** Jukka Jolkkonen, Rosalia Mendez-Otero, Gabriel Rodriguez de Freitas, Johannes Boltze, Paulo Henrique Rosado-de-Castro

**Affiliations:** ^1^Institute of Clinical Medicine-Neurology, University of Eastern Finland, 70210 Kuopio, Finland; ^2^Neurology, Neurocenter, University Hospital of Kuopio, 70210 Kuopio, Finland; ^3^Instituto de Biofísica Carlos Chagas Filho, Federal University of Rio de Janeiro, 21941-902 Rio de Janeiro, RJ, Brazil; ^4^D'Or Institute of Research and Education, 22281-100 Rio de Janeiro, RJ, Brazil; ^5^Fraunhofer Research Institution for Marine Biotechnology, 23562 Lübeck, Germany; ^6^Institute for Medical and Marine Biotechnology, University of Lübeck, 23562 Lübeck, Germany; ^7^Institute of Biomedical Sciences, Federal University of Rio de Janeiro, 21941-902 Rio de Janeiro, RJ, Brazil; ^8^Department of Radiology, Federal University of Rio de Janeiro, 21941-902 Rio de Janeiro, RJ, Brazil

Although cerebrovascular diseases are among the leading causes of health burden in the world, presently existing therapies have narrow capabilities in the treatment of such ailments [1, 2]. Cell therapies were originally used in hematological disorders and are currently being investigated as potential treatments for diverse conditions [3, 4]. Several preclinical reports have indicated that cell transplantation may generate beneficial functional and structural outcomes in stroke animals, even though the underlying mechanisms for such effects are still the subject of intensive research [5–7]. In the clinical setting, preliminary studies have been published indicating a good safety profile of systemic cell therapies, but additional trials are needed to assess the possible feasibility and efficacy of cell transplantation in cerebrovascular patients [5, 8, 9]. Furthermore, there are numerous obstacles to be tackled in order to thoroughly translate results from animal studies to patients [5, 8, 9].

This special issue includes reviews that provide insights into the current state of the art in cell therapies for cerebrovascular diseases. In one of the reviews, A. Nowakowski et al. discuss genetic engineering methods to improve the migration and survival of mesenchymal stem cells. In another review, L. S. B. Boisserand et al. debate the diverse uses and the physical and mechanical characteristics of biomaterials associated with cell transplantation in experimental ischemic stroke. In another review, P. H. Rosado-de-Castro et al. analyze characteristics of preclinical and clinical studies of bone marrow-derived cell therapy for hemorrhagic stroke models, such as cell dose, routes of cell delivery, and time window.

This special issue also features original articles investigating different aspects being of direct translational relevance for the study of cell transplantation in cerebrovascular lesions. L. Cui et al. conducted a flow cytometry-based pulse-width test to evaluate the consequences of diverse cell suspension concentrations, storage mediums, storage times, and freeze-thawing technique on the clumping of rat bone marrow-derived mesenchymal stromal cells, as well as cell viability. Unexpectedly, they found that increased cell concentrations did not lead to increased clumping* in vitro*. They also reported that fresh cells in normal saline had higher viability and less clumping than frozen-thawed cells.

A. Pikhovych et al. investigated the effects of transcranial direct current stimulation (tDCS) in the brain of mice. They reported that multisession anodal tDCS at low charge density downregulated cortical microglial constitutive expression of Iba1. On the other hand, anodal and cathodal tDCS increased neurogenesis in the subventricular zone.

B. Yang et al. investigated the influence of cryopreserving bone marrow mononuclear cells on their viability and on their effects in mice after middle cerebral artery occlusion. They reported that although cryopreservation had a negative impact on cell viability, both fresh and cryopreserved mononuclear cells had comparable behavioral and histological effects on the animal stroke model.

F. Moniche et al. carried out pooling data evaluation of two pilot clinical studies with autologous bone marrow mononuclear cell (BM-MNC) therapy in ischemic stroke subjects. They found a correlation between higher dose of BM-MNCs and better outcome as assessed by modified Rankin scale score of 0–2 at 6 months, mainly when more than 310 × 10^6^ cells were transplanted.

Finally, W.-H. Fang et al. characterized uptake and release of a p5-CDK5 inhibitory peptide by human adipose tissue-derived mesenchymal stem cells. They reported that the peptide was capable of blocking the CDK5 pathway, associated with apoptosis, indicating potential application for cell therapies after stroke.

In summary, significant findings have been made in cell therapy research for cerebrovascular diseases and the reviews and original articles in this special issue highlight developments and challenges for translation of such promising therapies into the clinic.

*Jukka Jolkkonen* *Jukka Jolkkonen* *Rosalia Mendez-Otero* *Rosalia Mendez-Otero* *Gabriel Rodriguez de Freitas* *Gabriel Rodriguez de Freitas* *Johannes Boltze* *Johannes Boltze* *Paulo Henrique Rosado-de-Castro* *Paulo Henrique Rosado-de-Castro*
